# Bulbar-Onset Amyotrophic Lateral Sclerosis Unmasked by General Anesthesia: A Case Report

**DOI:** 10.7759/cureus.84823

**Published:** 2025-05-26

**Authors:** Amine Bouabdallaoui, Salma Taouihar, Naji Yahya, Nawal Adali, Hicham Nassik

**Affiliations:** 1 Anesthesia and Critical Care, Ibn Zohr University, Faculty of Medicine and Pharmacy, Agadir, MAR; 2 Neurology, Ibn Zohr University, Faculty of Medicine and Pharmacy, Agadir, MAR

**Keywords:** amyotrophic lateral sclerosis, anesthesia, case reports, general surgery, neurology

## Abstract

Amyotrophic lateral sclerosis (ALS) is a progressive neurodegenerative disease that primarily affects the motor neurons in the brain and spinal cord, resulting in severe muscular weakness, atrophy, and loss of motor control. Patients with ALS are often highly sensitive to anesthetic drugs, specifically neuromuscular blocking agents, which can exacerbate muscle weakness and contribute to prolonged postoperative recovery times. In this article, we report a case of bulbar ALS diagnosed after postoperative extubation failure in a 51-year-old patient. Practitioners should consider this disease in cases of difficult postoperative ventilatory weaning and should be aware of the impact of surgery and anaesthesia on disease progression.

## Introduction

Amyotrophic lateral sclerosis (ALS) is a progressive neurodegenerative disease that primarily affects the motor neurons in the brain and spinal cord, resulting in severe muscular weakness, atrophy, and loss of motor control. ALS has an annual incidence of one to two per 100,000 individuals, with a higher prevalence in older populations, often manifesting in the fifth or sixth decade of life [1-2]. ALS can be classified by its onset type, including limb onset, which primarily affects limb mobility, and bulbar onset, which impairs cranial nerve function, leading to symptoms such as dysarthria, dysphagia, and eventual respiratory failure​ [3]. In particular, bulbar-onset ALS presents significant perioperative risks due to the involvement of respiratory and pharyngeal muscles, complicating airway management and increasing the likelihood of aspiration and respiratory compromise during general anesthesia (GA). Patients with ALS are often highly sensitive to anesthetic drugs, specifically neuromuscular blocking agents, which can exacerbate muscle weakness and contribute to prolonged postoperative recovery times [1-2]. We report a case of bulbar-onset ALS diagnosed after postoperative extubation failure in a 51-year-old patient. Practitioners should consider this disease in cases of difficult postoperative ventilatory weaning and should be aware of the impact of surgery and anaesthesia on disease progression.

## Case presentation

A 51-year-old male patient with a history of pulmonary tuberculosis treated in 1994 was admitted to a private clinic for a white-line hernia, for which he was operated on the same day under GA. Preoperative assessment revealed a conscious patient with no sensorimotor deficits, hemodynamic and respiratory stability, ASA 1, metabolic equivalent (MET)>4, no criteria for difficult intubation, and a palpable mass in the white line on abdominal examination. The patient underwent surgery under GA, induced with fentanyl, propofol, and rocuronium, and maintained with halogen (sevoflurane), with no reported incidents during the procedure. The patient was awakened and extubated immediately after surgery without requiring the administration of antagonist medications. The clinical course was complicated a few hours later by the development of respiratory distress requiring reintubation. Then, the patient was transferred to the intensive care unit of the tertiary-level hospital, intubated, ventilated, and sedated for additional care. 

Upon admission to our intensive care unit, the clinical examination revealed a patient weighing 80 kg, with a BMI of 29.3; the patient was already intubated, sedated with midazolam and fentanyl, pupils in miosis, hemodynamically stable with a noninvasive blood pressure (NIBP) 130/75 mmHg and heart rate (HR) 90 bpm, ventilated in volume-controlled mode with 100% pulsed oxygen saturation under an inspired oxygen fraction of 50%, and febrile with a temperature of 38.2 °C. An abdominal examination revealed an operative scar on the white line. An electrocardiogram showed a coronary sinus rhythm with HR at 75 bpm, abrasion of the R wave in the anterior territory, suspended ST segment in D1, flattened T wave in D3, and inverted T wave in V1. Cardiac Doppler ultrasound performed by cardiologists showed no abnormalities. Laboratory results are presented in Table 1.

**Table 1:** Laboratory test results

Laboratory tests	Results	Reference range
White blood cells	14930	4000-10000 (/mm^3^)
Hemoglobin	14.3	11-15 (g/dl)
Platelets	357000	150000-400000 (/mm^3^)
C-reactive protein	30	<6 (mg/l)
Procalcitonin	1.51	<0.5 (ng/ml)
Natriemia	138	136-145 (meq/l)
Kalaemia	3.86	3.5-5 (meq/l)
Calcaemia	90	85-105 (mg/l)
Magnesaemia	21	16-26 (mg/l)
Phosphaemia	37.3	25-45 (mg/l)
Uraemia	0.2	0.15-0.5 (g/l)
Creatininaemia	7.3	7-12 (mg/l)

**Figure 1:**
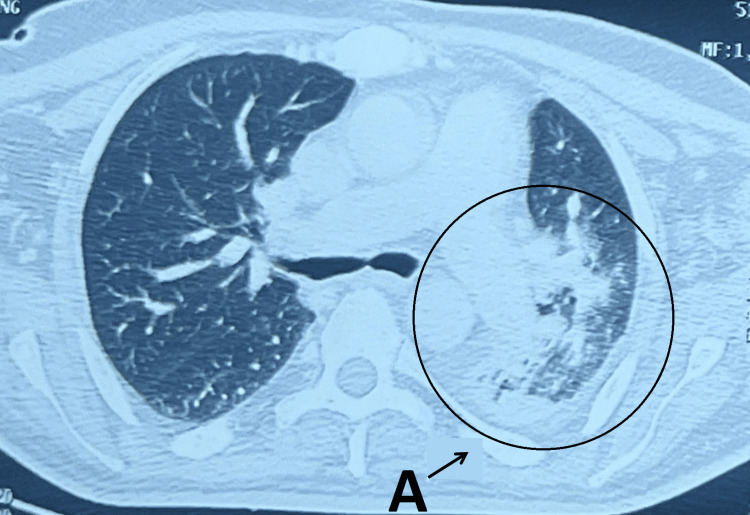
Thoracic CT scan revealing an image of left basal pneumonia A: image of left basal pneumonia

Chest radiography revealed left basal pneumonia. An injected thoracic CT scan revealed an image of left basal pneumonia suggestive of an infectious origin (Figure 1). The patient was treated with piperacillin-tazobactam (4g/0.5 g) administered as one vial every six hours after bacteriological sampling. Then, tracheostomy was performed a few days after antibiotic therapy was initiated due to difficult ventilator weaning. Rigid bronchoscopy revealed no tracheobronchial tree stenosis.

Given clinical, biological, and radiological improvements, sedation was discontinued. The evolution was marked by an early and calm awakening after sedation was stopped. The patient underwent a ventilatory weaning protocol with reinforced motor and respiratory physiotherapy and the placement of a silver cannula. The patient was subsequently decannulated due to respiratory and neurological improvement, and he remained hemodynamically and respiratorily stable with an oxygen saturation of 99% on ambient air. Seventy-two hours after decannulation, the patient presented with altered consciousness due to a hypercapnic coma, with a PaCO_2_ of 113 mmHg, requiring urgent intubation and recannulation, followed by mechanical ventilation. Thoracic imaging was performed, as well as a biological assessment that included an inflammatory profile and a blood electrolyte panel, including potassium, calcium, magnesium, and phosphate levels, all of which returned without abnormalities. Sedation was discontinued immediately after tracheal recannulation in the presence of a normal chest X-ray. The patient awoke rapidly, with a controlled PaCO₂ of 47 mmHg under spontaneous ventilation mode with inspiratory support.

**Figure 2:**
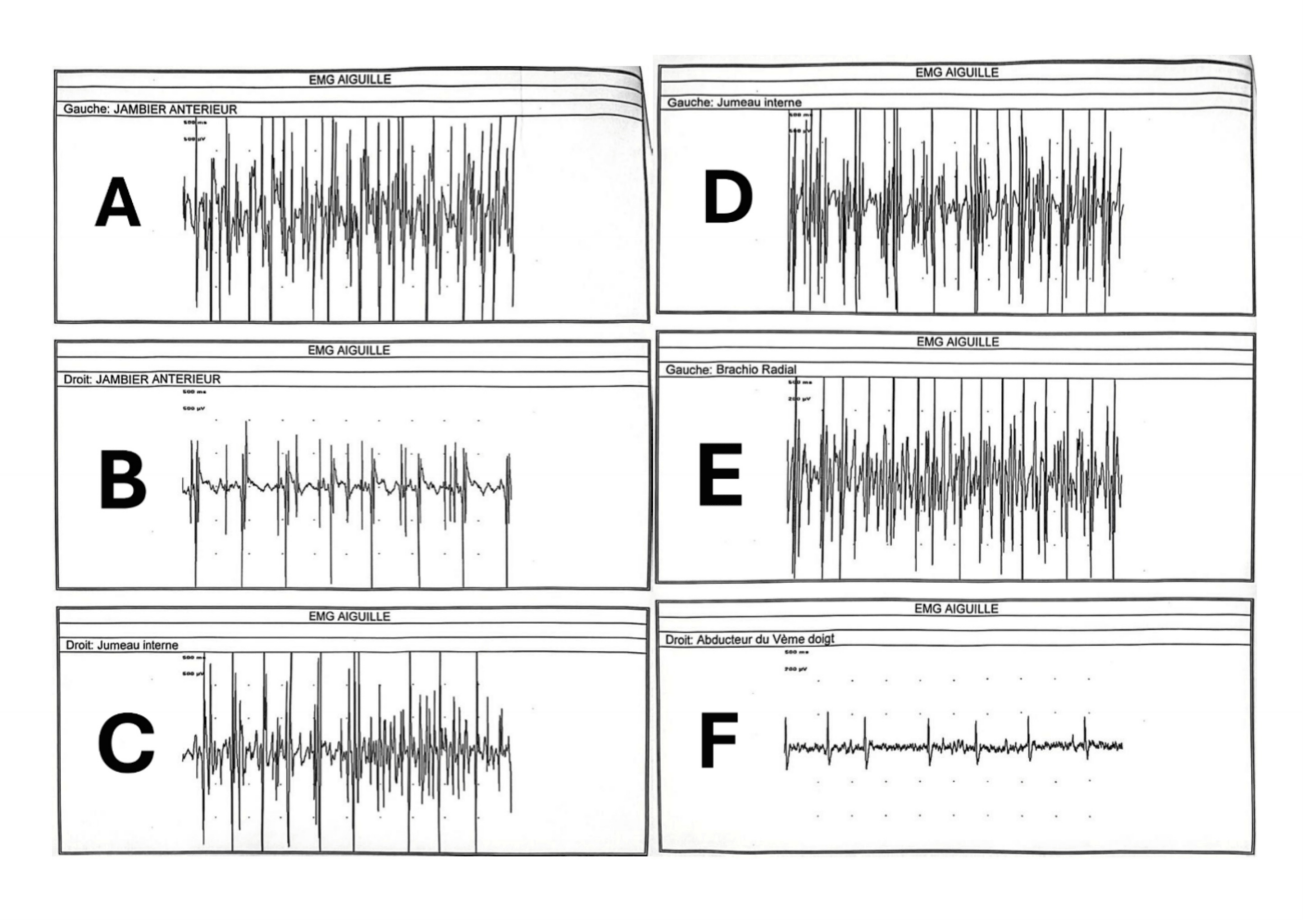
Needle EMG showing pure motor axonal affection, without alteration of sensory potentials but with signs of denervation detected in the four limbs suggesting involvement of the anterior horn A: Needle EMG of the left tibialis anterior muscle. B: Needle EMG of the right tibialis anterior muscle. C: Needle EMG of the right gastrocnemius muscle. D: Needle EMG of the left gastrocnemius muscle. E: Needle EMG of the left brachioradialis muscle. F: Needle EMG of the right fifth-digit adductor muscle.

A clinical evaluation by otolaryngologists and pulmonologists was requested to find an explanation for the patient's respiratory decompensation. Obstructive sleep apnea syndrome (OSAS) was initially considered but was later ruled out by pulmonologists due to the absence of clinical criteria. Due to the lack of explanation for the hypercapnic coma, a neurologist’s opinion was requested. Neurological assessment revealed hypotonia with motor weakness estimated to be 3/5 bilaterally in the upper limbs, along with lingual fasciculations and amyotrophy. Deep tendon reflexes were exaggerated in the four limbs. There was no truncal ataxia with no slow or hypermetric saccades. Cognitive assessment revealed mild deficits in attention. Electrophysiological studies (EMG) showed normal latency with low amplitude of motor potentials in both upper limbs, and sensory nerve conduction was within the normal range. Needle EMG confirmed signs of denervation in the tongue and upper limbs, suggesting anterior horn involvement (Figure 2). Cerebrospinal MRI showed no corticospinal tract hyperintensity, hypointensity in the motor cortex, or brain atrophy. Laboratory evaluation of complete blood cell count, biochemical tests (blood urea nitrogen, creatine, sodium, potassium, phosphocalcic, and glucose), serum protein electrophoresis, blood hepatitis B and C, HIV serology, antinuclear antibody titer, and thyroid tests were normal. The CSF study results were normal (cell count: four cells; protein at 0.40g/l). Bacterial and viral markers in the CSF were negative.

Based on the physical examination and electrophysiological study, a diagnosis of bulbar-onset ALS was made. The patient received riluzole 50 mg tablets, one tablet twice a day, and underwent a ventilatory weaning protocol, as well as reinforced respiratory and motor physiotherapy, along with a high-protein and normocaloric diet. The evolution was favorable, with ventilatory weaning and decannulation two months later. The patient was discharged from the hospital under medical treatment and non-invasive ventilation at home, with follow-up consultation by neurologists. 

## Discussion

ALS is an adult-onset neurodegenerative disease caused by the progressive death of central and peripheral motor neurons and is characterized to varying degrees by progressive motor symptoms such as progressive paralysis of the limbs, bulbar dysfunction (dysarthria, dysphagia), and restrictive respiratory failure due to respiratory muscle involvement [4]. It is a rare disease with an incidence ranging from two to three cases per 100,000 individuals per year in Europe. Data were not available for Morocco [5].

In 90% of cases, the disease is sporadic and related to a spontaneous mutation in one or more genes. In 10% of cases, it is familial, and the four genes most often involved are SOD1, C9ORF72, TARDBP, and FUS [5]. Although the pathophysiology of ALS is not well understood, the neuropathological hallmark of this disease is the aggregation and accumulation of ubiquitinated protein inclusions in motor neurons. The biological processes leading to the formation of these inclusions have been intensively studied but remain poorly understood [5].

Clinically, approximately 2/3 of people with classic ALS have a spinal onset form characterized by the development of amyotrophy and weakness of the lower and/or upper limbs. The most common telltale sign is impaired manual dexterity and/or walking. Spasticity frequently develops in these patients. People affected by the bulbar-onset form of the disease initially have dysarthria and dysphagia, which predominates in liquids. Manifestations in limbs may appear simultaneously or after a variable delay. Paralysis is progressive and can lead to respiratory failure due to diaphragmatic involvement, which is life-threatening. Other manifestations may be added to motor disorders, such as constipation, weight loss, pain, circulatory disorders (risk of phlebitis), and sleep disorders. Approximately 5% of people have signs of frontotemporal dementia, combining behavioral disorders with impairment of executive function and language [6].

In our case, the form was atypical, with primarily respiratory clinical manifestations revealed by immediate postoperative extubation failure after general anesthesia, followed by difficult weaning from mechanical ventilation over a period of five months. There is no test that provides absolute certainty for diagnosis. The diagnosis was based on a combination of positive and negative findings, patient history, and clinical examination, which were the most important elements. Certain functional explorations of the nervous system are useful, particularly the electroneuromyogram, which confirms motor neuron involvement, assesses its extent, and rules out diseases affecting motor nerves and muscles. Motor-evoked potentials confirm the involvement of upper motor neurons. All other tests, including blood tests, CT scans, MRIs, and lumbar punctures, aim to rule out other diseases that may resemble ALS. The diagnostic and prognostic roles of the neurofilament light chain are currently the subject of extensive research [6].

This case suggests acceleration of disease progression by surgery and general anesthetic drugs. In the literature, some studies have observed an acceleration of disease progression during the three-month period after surgery; the mechanisms of aggravation remain poorly understood and are likely related to the inflammatory response caused by surgical aggression [7]. Patients with ALS are extremely sensitive to nondepolarizing neuromuscular blockers. Studies available in the literature have shown a greater benefit of locoregional anesthesia in this type of patient; in the case of impossibility of locoregional anesthesia, general anesthesia without the use of nondepolarizing neuromuscular blocking agents is possible. As ALS is not a common disease, there are a limited number of publications in the literature [8]. If muscle relaxation is necessary, curarization monitoring is highly required [9-10]. The short duration of action of propofol makes it unlikely that a single infusion could significantly influence the pathophysiology of ALS [10-11]. As for opioids and halogenated agents, there are insufficient data in the literature regarding their impact on the progression of ALS.

Once ALS is diagnosed, multidisciplinary care is necessary. Although medications available to slow the progression of lateral sclerosis are limited, special attention to the patient's respiratory function, nutrition, and mobility can positively influence the course of the disease. Riluzole is the only approved medication that has demonstrated its potential impact on extending the survival of patients with ALS, but with a modest benefit and a high cost, which limits its prescription by some neurologists [12].

## Conclusions

Anesthetic management in patients with bulbar-onset ALS requires immediate and careful adjustment to avoid exacerbating respiratory complications. Evidence supports the use of regional anesthesia and total intravenous anesthesia (TIVA) without muscle relaxants as safer alternatives to general anesthesia, minimizing respiratory depression and the risk of postoperative complications. This case underscores the importance of multidisciplinary planning and vigilance in recognizing undiagnosed ALS symptoms in the perioperative setting, ensuring that anesthesia plans align with the patient’s unique respiratory and neuromuscular challenges.
